# Erythropoietin-Induced Changes in Bone and Bone Marrow in Mouse Models of Diet-Induced Obesity

**DOI:** 10.3390/ijms21051657

**Published:** 2020-02-28

**Authors:** Sukanya Suresh, Josue Caban Alvarez, Soumyadeep Dey, Constance Tom Noguchi

**Affiliations:** Molecular Medicine Branch, National Institute of Diabetes and Digestive and Kidney Diseases, National Institutes of Health, Bethesda, MD 20814, USA; suresh@iu.edu (S.S.); josue.caban7@gmail.com (J.C.A.); shoumo3@gmail.com (S.D.)

**Keywords:** erythropoietin, obesity, bone, bone marrow adipocyte, osteocyte, osteoblast, osteoclast, stromal cells, FGF23

## Abstract

Obesity remodels bone and increases bone marrow adipocytes (BMAT), which negatively regulate hematopoiesis and bone. Reduced BMAT could restore altered hematopoiesis and bone features. We analyzed the potential of erythropoietin (EPO), the cytokine required for erythropoiesis, to inhibit BMAT in C57BL6/J mice fed four weeks of a high-fat diet (HFD). Acute EPO administration markedly decreased BMAT in regular chow diet (RCD) and HFD-fed mice, without affecting whole body fat mass. Micro-CT analysis showed EPO reduced trabecular bone in RCD- and HFD-fed mice, but EPO-treated HFD-fed mice maintained cortical bone mineral density and cortical bone volume, which was reduced on RCD. Despite achieving similar increased hematocrits with BMAT loss in RCD- and HFD-fed mice treated with EPO, decreased bone marrow cellularity was only observed in RCD-fed mice concomitant with an increasing percentage of bone marrow erythroid cells. In contrast, in HFD-fed mice, EPO increased endothelial cells and stromal progenitors with a trend toward the normalization of marrow homeostasis. EPO administration increased c-terminal FGF23 and intact serum FGF23 only in HFD-fed mice. These data demonstrate the distinct EPO responses of bone and marrow in normal and obese states, accompanying EPO-induced loss of BMAT.

## 1. Introduction

In adults, bone marrow adipose tissue (BMAT) comprises 70% of bone marrow volume and accounts for 10% of total fat mass [1,2]. BMAT has distinct origin and function from white and brown adipocytes [3]. In the bone marrow, BMAT originates from a subset of bone marrow stromal cells (BMSCs) called skeletal stem cells, which are also the precursors of osteoblasts that form the mineralized bone matrix [4]. Conditions of excessive marrow adiposity, such as ageing [5], estrogen deficiency, osteoporosis [6], obesity [7], caloric restriction [8], radiation and chemotherapy [9] are associated with bone loss and skeletal fragility, suggesting an inverse relationship between BMAT and bone remodeling. Specifically, the role of obesity in skeletal health has been contradictory with early observations that were suggestive of the protective role of obesity in bone health [10]. Subsequent studies in obese women showed that, despite increased bone mineral density, they had reduced bone strength and increased fracture risk [11,12]. Similarly, men with increased visceral fat and BMAT have reduced cortical bone parameters and lower bone mechanical strength [13]. Studies in animal models have shown that obesity increases bone resorption by enhancing osteoclast activity and by suppressing osteoblast function. The increased secretion of proinflammatory cytokines, like interleukin 6 and tumor necrosis factor-α, by adipocytes in obese states disrupts the osteoclast–osteoblast balance, resulting in impaired bone remodeling and bone loss [14]. Besides impacting bone health, increased BMAT expansion negatively regulates hematopoiesis while blocking BMAT results in hematopoietic recovery [15]. Thus, in pathological conditions characterized by excessive fatty marrow, interventions that can reduce BMAT expansion could prevent the associated bone loss and promote hematopoiesis.

Erythropoietin (EPO), a hormone produced by the adult kidney, is required for erythropoiesis and acts by binding to the EPO receptor (EPOR) on erythroid progenitor cells. EPOR is also expressed by diverse non-hematopoietic cells, including adipocytes [16]. In the bone microenvironment, EPOR is expressed in BMSCs as well as in osteoblasts and osteoclasts that contribute to bone remodeling. EPO has context-dependent effects on bone and can result in either bone formation or bone loss. In fracture healing models, EPO administration promoted bone formation [17,18,19,20], while healthy mice administered EPO [21,22,23,24], as well as transgenic mouse models with high human EPO [23,24] had a reduced trabecular bone volume and BMAT. Endogenous EPO signaling is also essential to the maintenance of healthy bone and marrow, as mice with EPOR restricted to the erythroid tissue had a decreased trabecular bone volume and increased BMAT [24]. EPOR expression in BMSCs is essential for their differentiation to osteoblast and adipocyte lineages, as in vivo ectopic ossification assays using BMSCs lacking EPOR resulted in ossicles with less bone volume and more BMAT, while BMSCs from mice expressing high transgenic EPO resulted in a marked reduction in both bone volume and BMAT, indicating that the loss of endogenous EPO signaling shifted BMSC differentiation toward adipogenesis, while elevated EPO inhibited BMSC differentiation toward both bone and adipocytes [24].

In mice, the EPO treatment-stimulated increase in hematocrit is accompanied by a loss of fat mass in external fat depots, particularly in male mice [25,26,27]. However, EPO administration reduced BMAT and promoted bone loss in both male and female mice [24], underscoring the different origins of white fat pads and BMAT. Moreover, the expression of EPOR in adipocytes is essential in the regulation of body weight and fat mass, as mice with erythroid-restricted EPOR expression had increased body weight and fat mass [25] and EPO administration in these mice did not reduce fat mass. These mice also had increased BMAT [24]. Nevertheless, EPO administration in these mice decreased BMAT in both male and female mice similar to their wild-type littermates, indicating that EPOR expression in BMAT is not required for the EPO-mediated reduction of BMAT [24].

In mouse models of excessive fat accumulation induced by high-fat diet (HFD) consumption, EPO administration resulted in greater reduction in white adipose tissue in comparison with mice fed regular chow diet (RCD) [26] indicating heightened EPO response in reducing fat accumulation in obese states. In obese mice, EPO also improved glucose tolerance and reduced white adipose tissue inflammation and body fat mass/weight in male mice [28]. Previously, it was reported that expansion of BMAT induced by HFD feeding in both male and female mouse models was associated with trabecular bone loss while cortical parameters was unchanged in male and female mice with increased expression of adipogenic genes and a reduction in osteogenic genes [29]. Whether EPO regulates bone differently in obese states and can regulate specific bone cells like osteocytes, osteoclasts and periosteal osteoblasts are unexplored. Considering the potential of EPO in reducing BMAT expansion, this study determined if administration of EPO in diet induced obese mouse models could reduce BMAT expansion and thereby maintain bone and restore bone marrow cell populations. 

## 2. Results and Discussion

### 2.1. EPO Administration Reduces BMAT in Mice Fed Regular Chow or High-Fat Diet

**Figure 1:**
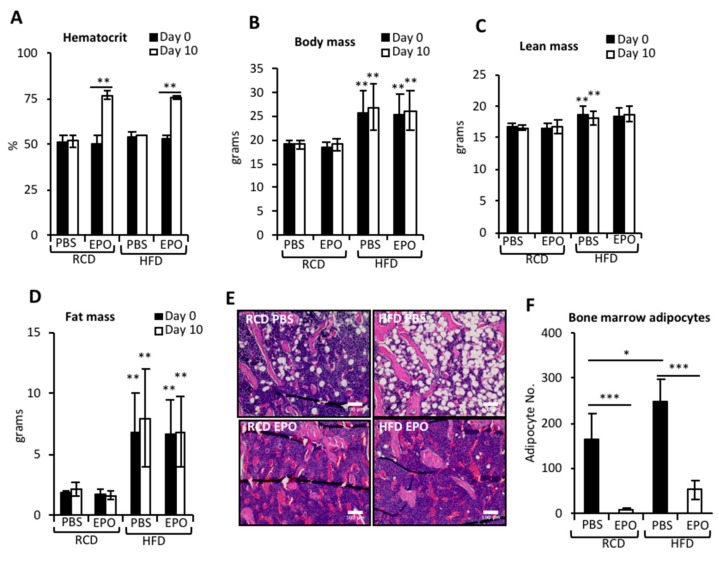
Erythropoietin (EPO) administration in high-fat diet (HFD)-fed mice decreases bone marrow adipocytes (BMAT) expansion. (**A**) The hematocrit measurements on day 0 (black bars) and day 10 (white bars) in female C57BL6/J mice fed either regular chow diet (RCD) or four weeks of high-fat diet (HFD) containing 60 kcal% fat (high-fat, 5240 kcal/kg, 34.9% crude fat). Blood was collected from tail in the heparin-coated capillary tubes, centrifuged and hematocrit was measured with a microhematocrit capillary tube reader (Veterinary Information Network Bookstore, Davis, CA, USA). Ten days of EPO treatment (1200 U/kg) increases hematocrit in both RCD and HFD-fed mice compared to the saline control. (**B**) The body mass after 4 weeks of RCD and HFD feeding in mice with and without ten days of EPO treatment. (**C,D**) The whole body composition was measured using the EchoMRI-100 system (Echo Medical Systems, Houston, TX, USA) to determine (**C**) lean mass and (**D**) adipose tissue mass in the external fat depots. (**E**) The representative H & E staining of the femurs of mice, showing adipocytes (white circular regions), scale 100 μm. (**F**) The enumeration of bone marrow adipocytes (*n* = 6/group). * *p* <0.05, ** *p* < 0.01, *** *p* < 0.001.

EPO treatment increased hematocrits in RCD- and HFD-fed mice similarly (Figure 1A). HFD feeding for 4 weeks resulted in significant increase in body weight and body fat mass (Figure 1B,D). An increase in lean mass was observed in both phosphate-buffered saline (PBS) and EPO-administered HFD-fed mice compared to the RCD-fed mice, however significance was achieved only in the comparisons between RCD–PBS and HFD–PBS (Figure 1C). Body weight, lean mass or fat mass was not affected by EPO treatment for 10 days (Figure 1B–D). BMAT, predominantly localized in the trabecular regions, increased after 4 weeks of HFD feeding (Figure 1E,F). Although EPO treatment did not reduce external fat mass in these mice (Figure 1D), EPO administration markedly reduced BMAT in both RCD- and HFD-fed mice (Figure 1E,F), indicating that the EPO response of BMAT is differentially regulated and more immediate compared with abdominal subcutaneous and visceral white adipose depots. We have previously observed that EPO treatment significantly reduced body weight and fat mass in male mice, but not in female mice. EPO treatment in ovariectomized female mice reduced fat mass, but estrogen supplementation in these mice prevented EPO-induced fat mass reduction [27]. Thus, in female mice, estrogen signaling interferes with the EPO response in the body fat. However, in the current study, EPO significantly reduced BMAT expansion in female mice, suggesting the lack of interference of estrogen signaling in the BMAT response to EPO.

### 2.2. EPO Administration Reduces Trabecular Bone but Maintains Cortical Bone in HFD-Fed Mice

**Figure 2:**
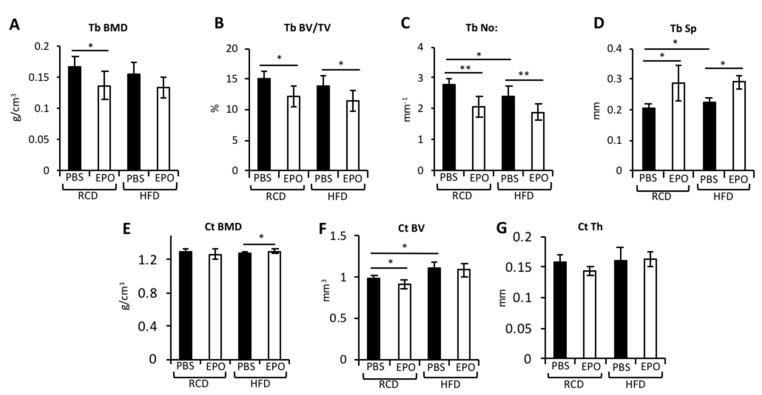
EPO administration in HFD-fed mice reduces trabecular bone volume and maintains cortical bone volume. (**A**–**G**) A Micro-CT quantification of the trabecular and cortical bone morphometry parameters. (**A**) The trabecular bone mineral density (Tb BMD), (**B**) trabecular bone volume/tissue volume (Tb BV/TV), (**C**) the trabecular number (Tb No:) and (**D**) trabecular spacing (Tb Sp), (**E**) the cortical bone mineral density (Ct BMD), (**F**) cortical bone volume (Ct BV) and (**G**) cortical thickness. *n* = 6 mice/group, * *p* <0.05, ** *p* < 0.01.

Micro-Ct analysis of the bones showed that EPO reduced the trabecular bone mineral density (BMD), trabecular bone volume/tissue volume, trabecular number and increased trabecular spacing in RCD-fed mice (Figure 2A–D). EPO did not alter cortical BMD in RCD-fed mice, but did diminish the cortical bone volume and showed a trend toward reduced cortical thickness (Figure 2E–G). HFD feeding resulted in a modest reduction in the trabecular number and increased their spacing, without altering the trabecular BMD and volume (Figure 2A–D). EPO treatment in HFD-fed mice behaved similarly to RCD-fed mice, with reduced trabecular bone volume/tissue volume, trabecular number and increased trabecular spacing, and a trend toward reduced trabecular BMD. However, HFD increased cortical bone volume, suggesting skeletal adaptation to withstand the increased body weight (Figure 2E–G). Unlike in RCD-fed mice, EPO administration in HFD-fed mice did not affect the cortical bone and maintained cortical bone volume and thickness with a marginal increase in cortical BMD. These data indicate differential EPO effects in the trabecular and cortical bones during obesity.

### 2.3. HFD Blunts EPO-Mediated Reduction of Cortical Osteocytes and Periosteal Osteoblasts

**Figure 3:**
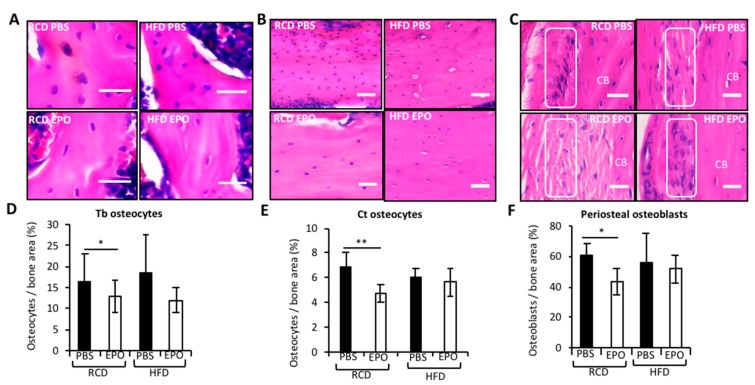
Histology analysis following EPO administration in RCD and HFD-fed mice. (**A**–**F**) The corresponding histology analysis of femurs. (**A**) The H & E staining of osteocytes (blue) embedded in trabecular bone (pink), scale 25 μm, (**B**) the cortical bone with osteocytes, scale 100 μm, (**C**) the periosteal region of bone containing osteoprogenitor cells adjacent to cortical bone (CB). Cells were counted within the region of interest in the periosteum indicated by the white box, scale 100 μm. (**D**) The enumeration of trabecular osteocytes, (**E**) cortical osteocytes and (**F**) periosteal osteoblasts. *n* = 6 mice/group, * *p* <0.05, ** *p* < 0.01.

Osteocytes are osteoblasts buried in the bone matrix that account for 95% of bone cells [30]. Osteoblast progenitors are the predominant cell type of periosteum surrounding cortical bones [31]. Bone histology analysis showed that, in RCD-fed mice, EPO reduced osteocytes in the trabeculae (Figure 3A,D) and cortical bones (Figure 3B,E), and osteoblasts in the periosteum (Figure 3C,F). HFD-fed mice did not exhibit any differences in these cell populations. In HFD-fed mice, EPO reduced trabecular osteocytes, but maintained cortical osteocytes and periosteal osteoblasts. These histology features are in accordance with the reduction in trabecular bone and maintenance of cortical bone with EPO treatment during HFD feeding.

### 2.4. EPO Administration Did not Affect Osteoclasts

**Figure 4:**
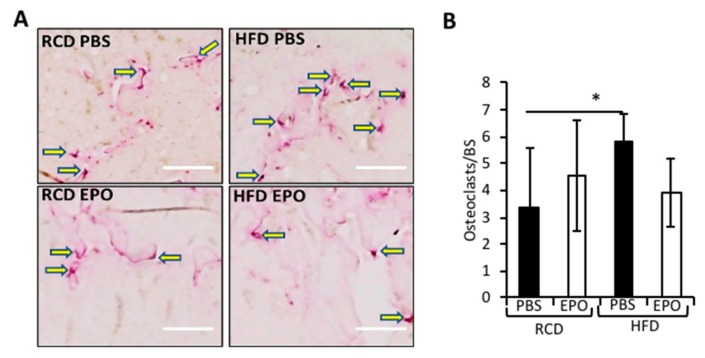
EPO administration does not affect osteoclasts in HFD-fed mice. (**A**) The titrate-resistant acid phosphatase (TRAP) staining of femurs showing osteoclasts with adjacent arrows, scale 50 μm, arrows indicate purple-colored osteoclasts. (**B**) The enumeration of osteoclasts per bone surface (BS) (*n* = 6/group), * *p* <0.05.

Osteoclasts, cells that resorb bone, were increased in HFD-fed mice (Figure 4A,B) as previously reported [32], and BMAT supports osteoclast differentiation in culture [33]. Published reports link EPO-stimulated bone loss to increased osteoclast numbers and activity. However, while EPO treatment reduced trabecular bone, we did not find that EPO treatment increased osteoclasts in RCD- or HFD-fed mice (Figure 4A,B). 

### 2.5. EPO Administration Restores Bone Marrow Cell Populations in HFD-Fed Mice

**Figure 5:**
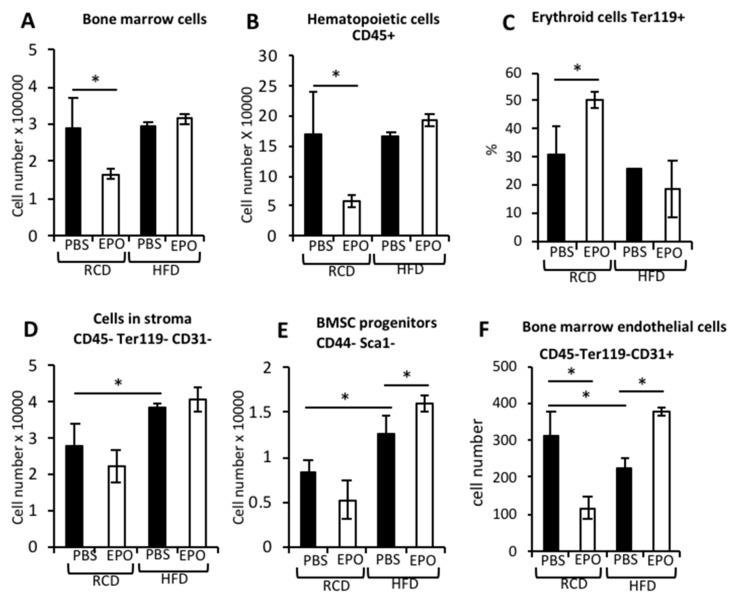
EPO administration in HFD-fed mice restores bone marrow cell populations. (**A**) The total number of bone marrow cells (minus red blood cells and adipocytes) analyzed by flow cytometry. (**B**,**C**) Flow cytometry analysis of bone marrow cells stained for the pan-hematopoietic marker CD45 (**B**) and erythroid specific glycophorin A, detected by a Ter119 antibody (**C**). (**D**) The total number of cells that are negative for CD45, Ter119 and CD31, considered as cells occupying bone marrow stroma. (**E**) The number of BMSC progenitors negative for CD44 and Sca1 in the CD45-Ter119-CD31- bone marrow stroma. (**F**) The number of bone marrow endothelial cells which are negative for the hematopoietic marker CD45, negative for erythroid marker Ter119 and positive for endothelin marker CD31. (*n* = 3-–6/group), * *p* <0.05.

EPO treatment in RCD-fed mice decreased BM (bone marrow) cellularity, CD45+ hematopoietic cells and endothelial cells (CD45-Ter119-CD31+), and increased percentage of Ter119+ erythroid cells (Figure 5A–F), consistent with the erythroid lineage bias reported for EPO and the suppression of bone marrow non-erythroid progenitors [34]. Although the increase in hematocrit with EPO treatment was comparable in RCD- and HFD-fed mice (Figure 1A), in HFD-fed mice with increased BMAT, EPO administration resulted in a greater decrease in adipocyte number compared with RCD (Figure 1F) and the decrease in BM cellularity and increase in the percentage of Ter119+ erythroid cells observed in RCD-fed mice were not evident (Figure 5A–F). The cell number in the BM stroma compartment (CD45-Ter119-CD31-) and the subset of BMSC progenitors (CD44-Sca1-) increased in HFD-fed mice (Figure 5D,E), suggesting the expansion of progenitors for enhanced marrow adipogenesis.

### 2.6. EPO Administration Increases Serum FGF23 Levels in HFD-Fed Mice

**Figure 6:**
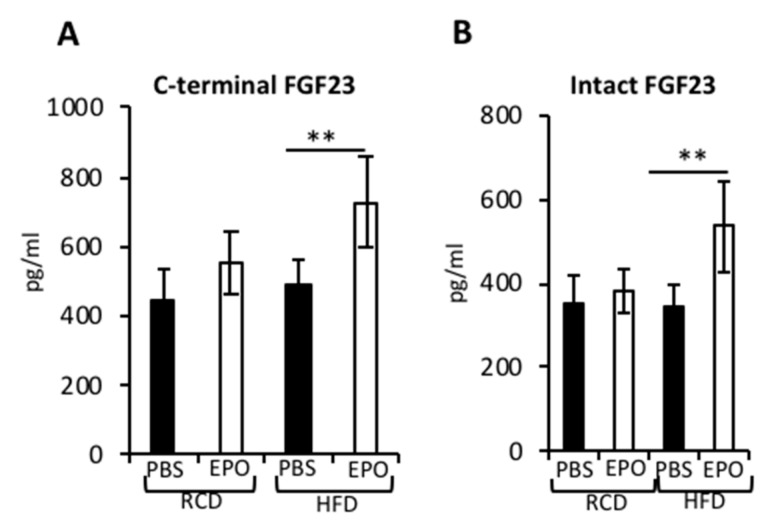
EPO-induced serum FGF23 in HFD-fed mice. (**A**,**B**) The serum levels of FGF23 measured using ELISA assays, (**A**) c-terminal FGF23 and (**B**) intact FGF23. (*n* = 3–6/group). * *p* < 0.01.

FGF23 is an osteocyte-derived hormone that regulates phosphate and vitamin D3 metabolism in the kidneys [35]. In humans and in mouse models, administration of EPO-stimulated FGF23 [36,37] and increased FGF23 subsequently decreased erythropoiesis [38]. In the current study, we found that in the RCD-fed mice, EPO administration showed a trend for increased c-terminal FGF23 (inactive form), but did not stimulate intact FGF23 (active form) (Figure 6A,B). In the HFD-fed mice, EPO administration significantly increased both c-terminal FGF23 and intact FGF23. With increased intact FGF23, there was suppression of the percentage of Ter119+ erythroid cells in the bone marrow of HFD-fed mice that received EPO (Figure 5C).

## 3. Discussion

In this study, we evaluated the effects of EPO administration on BMAT, trabecular and cortical bone, as well as on the bone marrow cell population under HFD feeding conditions in female C5BL6/J mice. HFD consumption for 4 weeks resulted in significant BMAT induction in the marrow with localization of adipocytes predominantly in the trabecular cavities of the bone. Even though trabecular bone comprises only 20% of the total bone, it is metabolically more active and has a faster bone remodeling rate than cortical bone [39]. The presence of BMAT near trabecular bone regions indicates a more active role for BMAT in its remodeling. Trabecular bone is also more responsive to stimuli and, with 4 weeks of HFD, we observed reduced trabecular number and increased trabecular spacing. However, with this duration of HFD, there was no significant change in the bone mineral density and trabecular bone volume. Prolonged HFD feeding might have accentuated these effects and affected the net bone volume and density of trabecular bone. With ten-day EPO administration, there was a significant reduction in BMAT, while total body fat mass was unaffected. This shows the heightened response of BMAT to EPO compared to the adipocytes in visceral and subcutaneous depots. EPO is known to decrease preadipocyte differentiation [25] and increase the oxidative metabolism and fatty acid oxidation of white adipocytes [26]. Elevated EPO also reduces the expression of Ppar-γ in BMSCs, thus contributing to reduced bone marrow adipogenesis [24]. Similar mechanisms might be responsible for EPO-mediated reduction in BMAT.

We report that both HFD feeding and EPO administration have distinct effects on trabecular and cortical bone. With the reduction in trabecular number and the increase in trabecular spacing, there was a concomitant increase in cortical bone volume and thickness, suggesting a skeletal adaptation to mechanical loading with increased body weight. In an earlier study which addressed BMAT expansion, C57BL6/J females with six weeks of 45 kcal% HFD feeding did not exhibit any changes in trabecular or cortical bone [40]. The HFD-induced bone changes observed in our study could be due to the consumption of a diet with a higher fat content of 60 kcal%. With EPO administration, the trabecular bone reduction was similar to that seen in RCD-fed mice. However, EPO-induced reduction in cortical bone volume and thickness was seen only in RCD-fed mice. The lack of cortical bone reduction in HFD-fed mice with EPO suggests a blunted response to EPO under obese conditions, leading to the maintenance of cortical bone needed for accommodating the mechanical stress due to increased body fat mass.

Osteocytes are the longest living cell type in the bone, they develop dendritic processes known as canaliculi networks, which are essential for cell communication. Osteocytes are responsive to the mechanosensory adaptation of the skeleton [41] and the secretion of sclerostin and receptor activator of nuclear factor κB ligand (RANKL) by osteocytes regulate osteoblasts and osteoclasts, respectively [42,43]. The significance of osteocytes in regulating hematopoiesis was observed in mouse models lacking osteocytes, in which both B and T lymphopoiesis were severely impaired [44]. We observed that, with EPO administration, the number of osteocytes in trabecular bone and cortical bone are reduced in RCD-fed mice. However, in HFD-fed mice, only the number of osteocytes in trabecular bone are reduced. The maintenance of cortical osteocytes in HFD-fed mice, even with EPO administration, might be associated with the absence of EPO-dependent cortical bone reduction in these mice. We also observed a novel role for EPO in regulating periosteal osteoblasts that might also be contributing to the maintenance of cortical bone in HFD-fed mice. Periosteum is a membranous structure that surrounds the bone and consists of an inner cambial layer adjacent to the cortical bone surface that is rich in osteoblast progenitors, which can differentiate into osteoblasts, and an outer fibrous layer containing fewer fibroblasts in a collagenous matrix [31]. Periosteal expansion is associated with promoting bone strength and in conditions of bone loss, such as estrogen deficiency in postmenopausal women [45]. In rodent models limited periosteal expansion is associated with slender and fragile bones [46]. In our study, HFD itself did not affect periosteal osteoblasts, although EPO regulation of these cells was diet dependent, as the reduction in these cells was observed with RCD and not with HFD.

Increased BMAT in HFD-fed mice did not affect the bone marrow hematopoietic cell population. However, the reduction in bone marrow cellularity seen with EPO treatment in RCD-fed mice was not seen in HFD-fed mice, suggesting that the reduction in BMAT with EPO treatment in HFD-fed mice may maintain hematopoietic proliferation in bone marrow along with stimulated erythropoiesis. The increase in BMSCs was consistent with the previous report of increased stromal cells in diet-induced obese mice [47]. EPO-treated RCD-fed mice exhibited a trend for reduced stroma and BMSC progenitors and decreased BM CD31+ (endothelin) endothelial cells. In comparison, in HFD-fed mice, EPO did not reduce stroma and increased BMSC progenitors. Since BMSC progenitors can develop into osteoblasts, this increase in BMSC progenitors with EPO in HFD-fed mice might support the need for bone formation to achieve skeletal homeostasis. While HFD feeding lowered BM endothelial cells, EPO treatment in HFD-fed mice increased BM endothelial cell numbers to a level similar to that of control RCD-fed mice. Obese individuals have reduced BM endothelial cells, which is proposed to reduce vasculature [48]. In the bones, osteoblast precursors reach bone formation sites by moving through proximal blood vessels [49]. Therefore, an increase in BM endothelial cells in EPO-treated mice on HFD feeding could increase vasculature, leading to bone repair.

We assessed serum levels of intact FGF23 and c-terminal FGF23 with EPO administration in RCD and HFD-fed mice, as EPO-FGF23 is a key signaling pathway regulating the kidney–bone axis. In humans, serum levels of FGF23 increase with higher body mass index and fat mass, and FGF23 levels were higher in individuals with metabolic syndrome [50]. In mouse models, HFD feeding significantly increased FGF23 that was dependent on upregulated tumor necrosis factor-α signaling [51]. In RCD-fed mice, we observed a trend for an increase in the c-terminal FGF23 and no changes in intact FGF23 levels, but in HFD-fed mice, serum levels of both c-terminal and intact FGF23 were significantly elevated. Previous studies in humans have reported EPO administration increasing c-terminal FGF23, while, in mouse models, c-terminal FGF23 increased after 24 h of EPO treatment, followed by increased intact FGF23 after 4 days [38]. In mouse models on iron supplementation, EPO increased both c-terminal and intact FGF23 within three days of EPO administration [37], with serum c-terminal FGF23 levels rising to 6000 pg/mL. In our study, we used the same strain of C57BL/6 mice of similar ages and we observed that, in EPO-treated mice, c-terminal FGF23 levels increased to 553 ± 90.3 pg/mL, which was much lower than previously reported levels of c-terminal FGF23 with EPO administration. Differences in the EPO dose used in these studies and, possibly, the iron status of the animals might be contributing to these differences. An absence of FGF23 increase with elevated EPO has also been reported in humans [52], suggesting a complex link between EPO and FGF23. While excess FGF23 is associated with impaired bone mineralization [53], in the RCD-fed mice, EPO-induced reduction in trabecular bone was independent of changes in FGF23. Furthermore, in HFD-fed mice, EPO increased both c-terminal and intact FGF23, but the cortical bone in these mice was preserved. The induction of serum FGF23 by EPO is counterbalanced by an increase in the intracellular cleavage of the protein, resulting in increased c-terminal FGF23. This balance in maintaining serum FGF23 levels was not observed with EPO administration in regular diet feeding.

In conclusion, EPO inhibits BMAT in RCD-fed mice and prevents excessive BMAT accumulation in diet-induced obesity, thus preventing the conversion to fatty marrow. Our data shows that EPO has distinct effects on trabecular and cortical bone in normal and obese conditions. EPO administration increases BMSC progenitors and bone marrow endothelial cells and restores hematopoietic and non-hematopoietic subsets of cells that are altered during obesity. Within the duration of our study, these positive responses elicited by EPO in bone marrow did not benefit the metabolically active trabecular bone, but resulted in the maintenance of cortical bone.

## 4. Materials and Methods

### 4.1. Mice, High-Fat Diet Feeding and EPO Treatment

Twenty-four six-week-old female C57BL6/J mice (Jackson Laboratories) were fed regular chow NIH07 diet (RCD) (Zeigler Brothers, Gardners, PA, USA) or high-fat diet (HFD) with 60 kcal% calories from fat (Research Diets, New Brunswick, NJ, USA) for four weeks. Mice were administered either saline or human Epoetin alpha subcutaneously at a dose of 1200 IU/kg daily for the last 10 days (Epogen) (Amgen, Thousand Oaks, CA, USA). Control mice were injected with phosphate buffered saline (PBS). Animal protocols followed National Institutes of Health guidelines and were approved by the National Institute of Diabetes and Digestive and Kidney Diseases animal Care and Use Committee (ASP K085-MMB-15, approval 11/09/2015; ASP K085-MMB-18, approval 11/09/2018).

### 4.2. Micro-CT Analyses

Femurs were prepared and scanned at 6 μm resolution using a Skyscan 1172 micro-CT scanner, as previously described [24]. Briefly, for trabecular and cortical bone morphometry, regions that are 0.35 and 4.25 mm from the growth plate, consisting of 1.5 mm sections of femur bone, comprising 257 bone slices, were individually analyzed using CTAn software.

### 4.3. Histology

Femurs were fixed in 10% buffered neutral formalin for 24 h followed by decalcification in 10% EDTA (0.5 M). Hematoxylin–Eosin (H&E) staining was performed on 6 μm sections. The number of osteocytes in trabecular and cortical bone and periosteal osteoblasts were counted and expressed in terms of bone area. For osteoclast detection, tartrate-resistant acid phosphatase (TRAP) staining was performed using a TRAP/ALP stain kit (FUJIFILM Wako Chemicals USA, Richmond, VA, USA) according to the manufacturer’s instructions. Purple-colored osteoclasts containing more than three nuclei were counted and the number of osteoclasts was normalized to the bone surface. Three fields were counted for each sample.

### 4.4. FACS Analysis

The bone marrow cells (1 × 10^7^), after erythrocyte lysis, were incubated with 0.5 μg of anti-mouse CD16/CD32 antibody for blocking Fc receptors. For analyzing BMSCs, bone marrow endothelial cells and erythroid populations, cells were incubated with CD45-APC, Ter119-APC-780, CD31-FITC, CD44-PE, Sca1+ APC-700 for 30 min, followed by washing in staining buffer, and were analyzed in FACS Calibur (BD Bioscience).

### 4.5. ELISA

Blood was collected terminally by cardiac puncture and levels of c-terminal FGF23 and intact FGF23 were measured using ELISA assays according to manufacturer’s instructions (Immutopics, San Clemente, CA, USA).

### 4.6. Statistical Analysis

Data are expressed as the mean ± standard deviation. Comparisons among multiple groups were assessed using one-way analysis of variance with Dunnet’s multiple comparison post hoc tests at α = 0.05 (Graphpad Prism 6).
